# Clinical Outcomes of Osteoarticular Extracorporeal Irradiated Autograft for Malignant Bone Tumor

**DOI:** 10.1155/2020/9672093

**Published:** 2020-03-30

**Authors:** Satoshi Takenaka, Nobuhito Araki, Takafumi Ueda, Shigeki Kakunaga, Yoshinori Imura, Ken-Ichiro Hamada, Hidetatsu Outani, Norifumi Naka, Akira Myoui, Hideki Yoshikawa

**Affiliations:** ^1^Department of Orthopaedic Surgery, Osaka University Graduate School of Medicine, 2-2 Yamadaoka, Suita, Osaka 565-0871, Japan; ^2^Department of Orthopaedic Surgery, Ashiya Municipal Hospital, 39-1, Asahigaoka, Ashiya 659-8502, Hyogo, Japan; ^3^Department of Orthopaedic Surgery, National Hospital Organization Osaka National Hospital, 2-1-14, Houenzaka, Chuo-ku, Osaka 540-0006, Japan; ^4^Department of Orthopaedic Surgery, Osaka International Cancer Institute, 3-1-69, temae, Chuo-ku, Osaka 541-8567, Japan; ^5^Medical Center for Translational Research, Osaka University Hospital, 2-2 Yamadaoka, Suita, Osaka 565-0871, Japan

## Abstract

**Methods:**

We retrospectively reviewed 33 patients who underwent osteoarticular ECIA after bone tumor resection from 1988 to 2014. We investigated complications, radiographic changes by the International Society of Limb Salvage graft evaluation criteria, and functional outcomes according to the Musculoskeletal Tumor Society scoring system.

**Results:**

Fifteen patients were reoperated upon due to infection (*n* = 9), protruding fixation implant (*n* = 4), or fracture of the grafted bone (*n* = 2). The average radiographic evaluation score was 66.4%, and the median functional score was 23 (77%). The radiographic score for the proximal humerus or proximal tibia was lower than that for the other locations. The functional score was not different among the autograft sites but was related to the radiographic score.

**Conclusion:**

Although osteoarticular ECIA is one of the reasonable surgical options for patients with tumors for which reliable prostheses are not available, we do not recommend osteoarticular ECIA as a routine procedure because of high complication rate.

## 1. Introduction

Reconstructive procedures after wide resection of malignant bone tumors include endoprosthetic replacement, allograft, recycled autograft, composite arthroplasty, and distraction osteogenesis. Recycled autograft involves reimplantation of resected bone treated with irradiation [1], liquid nitrogen [2], pasteurization [3], or autoclaving [4]. Since 1988, we have performed extracorporeal irradiated autograft in >100 patients. Recycled autograft has some advantages over endoprosthetic replacement, such as preserving bone stock, no problems with prosthetic wear, low cost, adapting to any location, preserving soft tissue attachment, and preserving the growth plate in the surrounding healthy bone. Recycled autograft also has advantages over allografts including having no risk of viral transmission or immunologic reaction, a precise anatomical fit, and the correct reattachment of tendons and ligaments, although ECIA is difficult to use in patients with severe bone resorption or bone deformity.

Recycled autograft after intercalary resection has had promising outcomes [5–7]; however, a few studies have reported a high incidence of graft failure in osteoarticular recycled autografts compared to intercalary recycled autografts. The complications as well as the radiographic and functional results for osteoarticular recycled autografts are not well documented. In particular, there are no reports on the difference in outcomes among graft sites. Thus, we investigated the complications, long-term radiographic findings, and functional results of osteoarticular ECIA and examined the difference in outcomes among graft sites in order to effectively utilize this technique.

## 2. Materials and Methods

After obtaining approval from our institutional review board, we retrospectively reviewed the medical records of 33 patients (median age, 17 years (6–67)) who underwent osteoarticular ECIA for reconstruction after resection of malignant bone tumors including osteosarcoma, Ewing sarcoma, chondrosarcoma, bone metastasis, or soft tissue sarcoma invading bone in our hospitals from 1988 to 2014. The patient characteristics are shown in Table 1. Median follow-up time was 125 months (range, 5–264). The resected bones were irradiated with a single dose of 50 Gy; however, 80 Gy was used in 2 patients with chondrosarcoma because it is radioresistant. The irradiated bones were grafted and fixed with plates (*n* = 20), intramedullary nails (*n* = 10), or screws and wires (*n* = 3).

We investigated the survival, recurrence, complications requiring reoperation, graft status, and radiographic evaluation according to the International Society of Limb Salvage (ISOLS) graft evaluation criteria (Table 2) using final X-rays that were obtained during the follow-up. For patients who underwent graft removal, radiographic evaluation was performed using the last X-ray obtained before graft removal. Because three patients did not have sufficient radiographic data to allow for evaluation of the graft according to the ISOLS radiographic scoring system, thirty patients with available radiographic data were selected for radiographic evaluation. We evaluated functional outcome in 21 patients who still had osteoarticular grafts at the time of the survey using the Musculoskeletal Tumor Society (MSTS) functional score.

All statistical analysis was performed using R version 3.2.2 with *P* values of <0.05 being considered as statistically significant. Categorical variables were compared using the Fisher exact test and continuous variables with the Mann–Whitney *U* test. Spearman's rank correlation coefficient was used to determine the relationships between outcome measures.

## 3. Results

Of the 33 patients, 22 were continually disease-free (CDF), 3 showed no evidence of disease (NED), 1 was alive with disease (AWD), and 7 had died of disease at the time of the survey. Local recurrence was not observed in any patient.

**Figure 1:**
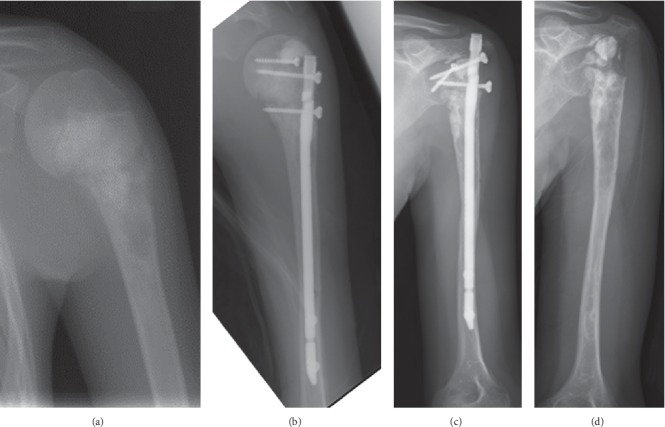
Case #6: 12-year-old female with osteosarcoma of the left proximal humerus. (a) Preoperative X-ray. (b) Immediately after treatment with osteoarticular extracorporeal irradiated autograft. (c) 14 years after operation, collapse of the graft and protrusion of the IM rod proceeded. (d) IM rod was removed.

**Figure 2:**
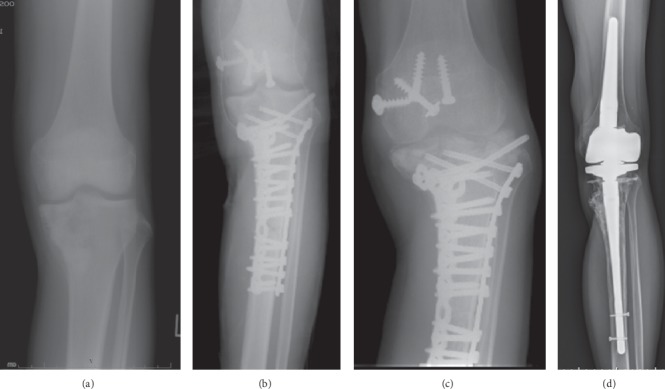
Case #27: 17-year-old male with osteosarcoma of the left proximal tibia. (a) Preoperative X-ray. (b) Immediately after treatment with osteoarticular extracorporeal irradiated autograft. (c) 16 months after operation, collapse of the autograft occurred and (d) reoperation with total knee arthroplasty composite with the remaining autograft.

**Figure 3:**
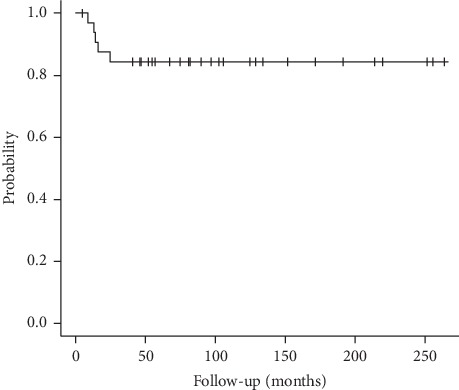
A survival curve of osteoarticular extracorporeal irradiate autograft in this study.

In total, fifteen patients (45%) had complications requiring reoperation at the primary site (Table 1). Infection occurred in 9 of 33 cases. In 5 of 9 cases, the infection was wound complication and/or soft tissue necrosis which were treated by debridement of soft tissue in most cases, but muscle cutaneous flap was needed in one acetabular case. The ECIA remained in these 5 cases. In the other 4 cases, deep infection occurred and we needed removing infected ECIA bone including articular surface. We performed 2nd stage revision using prosthesis in 2 cases, and nonvascularized fibula graft in one case, and cement spacer in one case. All infections occurred within 25 months after primary surgery. The number of surgeries before healing infection was 1 in four cases, 2 in four cases, and 3 in one case. Four cases of protruding implant required removal of the implant. Some of these cases were associated with bone resorption of the osteoarticular graft (Figure 1). Bone fracture occurred in 1 case of the distal humerus that was fixed with plate and iliac bone graft but remained nonunion. Aseptic collapse of the osteoarticular graft occurred in 1 case involving the proximal tibia, requiring removal of the articular surface of the graft and revision of the remaining autograft-prosthetic composite TKA (Figure 2). ECIA removal involving the articular surface was performed in 5 patients within 25 months of the first operation, although there were no cases in which ECIA was removed after the 25th month (Figure 3). In patients who underwent ECIA of the proximal tibia, the reoperation rate was quite high (83%), and the graft removal rate was significantly higher than removal in the other locations (*p*=0.031) (Table 3). Notably, the fixation method was not deemed to be associated with the complication rate.

**Figure 4:**
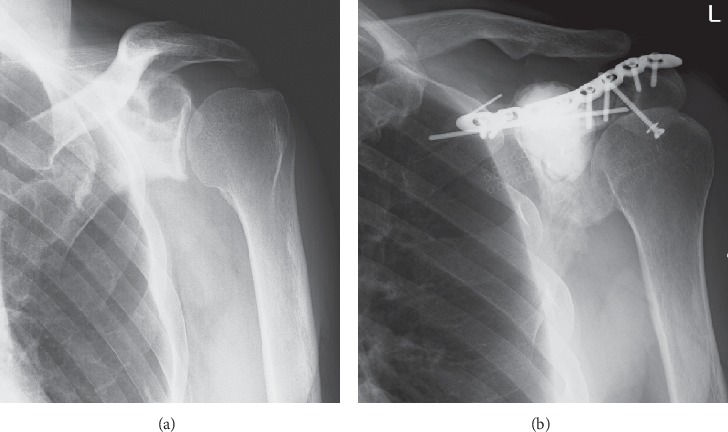
Case #3: 40-year-old male with grade 2 chondrosarcoma of the left scapula. (a) Preoperative X-ray and (b) 4 years after treatment with osteoarticular extracorporeal irradiated autograft.

The mean ISOLS radiographic evaluation score was 66.4% (range, 54.5%–97.9%). The proximal humerus and proximal tibia had significantly lower scores compared to the other locations in the categories of resorption (*p*=0.044), subluxation (*p*=0.0023), joint narrowing (*p*=0.012), subchondral bone (*p*=0.012), and total score (*p*=0.0087) (Figures 1 and 2) (Table 2). The scapula scored significantly higher compared to the other locations in the categories of resorption (*p*=0.0074), subluxation (*p*=0.020), joint narrowing (*p*=0.037), subchondral bone (*p*=0.0087), and total score (*p*=0.0093) (Figure 4). No significant relationship was observed between the fixation method and the radiographic evaluation score.

The average MSTS score was 73.8% (53.3%–93.3%). No significant difference in the MSTS score among graft sites was observed (Table 3). A positive relationship was identified between the MSTS functional scores and the ISOLS radiographic evaluation scores (*r* = 0.598, *p*=0.0042).

**Figure 5:**
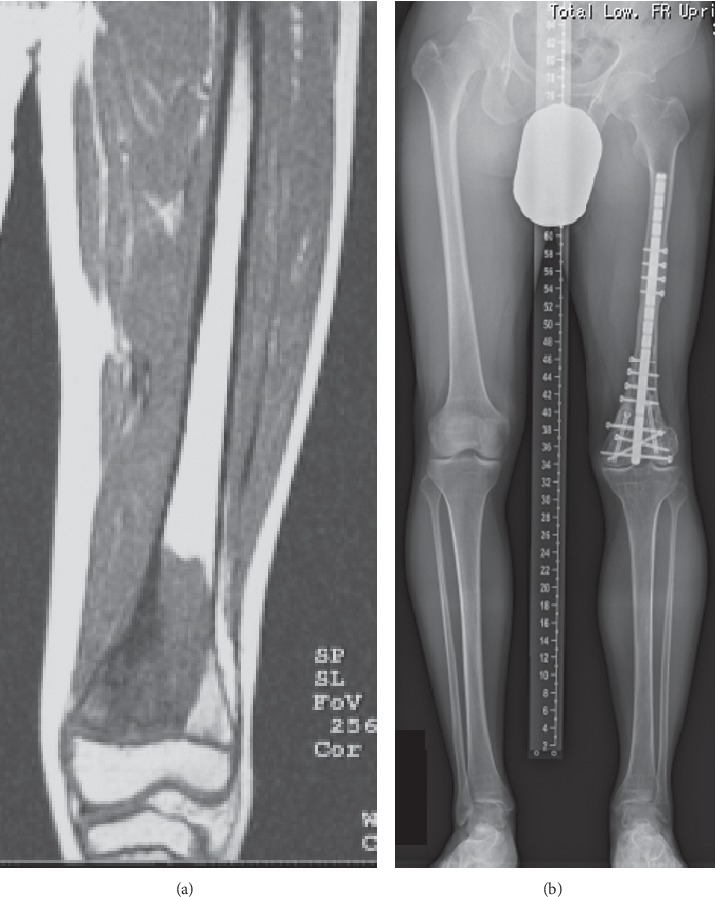
Case #23: 13-year-old male with osteosarcoma of the left distal femur. (a) Preoperative MRI. (b) 21 years after treatment with osteoarticular extracorporeal irradiated autograft. Collapse of the graft was not observed, and he did not undergo reoperation. Growth of the left tibia is preserved. The length of the left and right tibia is similar. The leg length discrepancy is 57 mm.

This study included 12 children patients under the age of 13. We analyzed if the children had better ISOLS radiographic evaluation score or MSTS functional score than adults. There were no significant differences, but the children under the age of 13 tended to have better MSTS score than the others (*p*=0.052). Four children patients with tumor around the knee still had the osteoarticular ECIA at the time of the survey (Cases #22, #23, #28, and #29). In all the 4 patients, growth of the surrounding healthy bone was preserved. For example, intact growth of the tibia was observed in those with distal femoral autograft (Figure 5). Percutaneous epiphysiodesis using transphyseal screws of the contralateral distal femur and the proximal tibia was performed in a single patient 5 years after the primary operation in order to minimize the discrepancy in leg length (Case #22). In these four patients, the mean discrepancy in leg length at the final follow-up was 38 mm (32–57 mm) with acceptable functional outcomes (MSTS score: median, 23.5 from 23 to 28).

## 4. Discussion

The treatment of primary bone malignancies has evolved significantly over the past 3 decades resulting in increased survival rates. Limb salvage surgery with endoprosthetic replacement is a promising treatment method with rapid mobilization. However, aseptic loosening, wear, and breakage are long-term concerns. Advantages of limb salvage surgery with ECIA include the incorporation of the host bone-graft, better longevity, precise fit into the bone defect, and preservation of soft tissue attachments. In fact, ECIA after intercalary resection is associated with good outcomes [5–7]. However, clinical outcomes of osteoarticular ECIA have not been elucidated. In this study, we demonstrated a high reoperation rate of this method and a significantly high removal rate in the proximal tibial ECIA, and bone resorption of the proximal humeral ECIA and proximal tibial ECIA was significantly observed than that of other osteoarticular ECIAs.

Our study has a number of limitations. Firstly, the limited number of patients hindered us in our retrospective study. Therefore, it was not possible to entirely evaluate the risk factors for complications and clinical outcomes. In addition, some inherent heterogeneity in terms of chemotherapy and type of internal fixation in our study could affect the incidence of complications and graft survival.

The complication rate for ECIA including intercalary, osteoarticular, or composite graft is reported between 12% and 45% [5–12] (Table 4). In this study, which is limited to the osteoarticular extracorporeal irradiated autograft, the rate of complication requiring reoperation was high (45%, 15/33). We speculate that osteoarticular ECIA is vulnerable to infection or fracture compared with other ECIAs because revascularization in osteoarticular ECIA occurs only from one side of autograft. The high complication rate of the osteoarticular graft was also reported in the frozen autograft treated with liquid nitrogen. Igarashi et al. reported a graft removal rate of 43.7% (7/16) for frozen osteoarticular autograft, which is comparable to or higher than our result [13], while Wu et al. reported no difference in union rate, incidence of complications, or graft failure between ECIA and liquid nitrogen-treated autografts [12]. In general, the complication rate for the osteoarticular recycled autograft is quite high. In particular, we observed significantly high removal rate in patients with proximal tibia osteoarticular ECIA (Table 2). In allograft, a high complication rate for proximal tibia osteoarticular allograft has also previously been reported [14,15]. Poor soft tissue coverage was considered to be a major reason for the higher risk in the proximal tibia. Due to the high complication risk, we do not recommend osteoarticular ECIA as a routine procedure, particularly for the proximal tibia.

In the previous study [6, 9, 11, 12, 16], the mean ISOLS radiographic evaluation score for ECIA was reported to be 74%–80.5% and it was 66.4% (range, 54.5%–97.9%) in the present study. Compared with the previous study, the lower score in our study is probably because it is limited to osteoarticular grafts. We observed a difference in ISOLS radiographic score among the graft sites (Table 2); the score was lower in the proximal humerus or the proximal tibia and higher in the scapula. Avascular necrosis, bone resorption, and subchondral bone collapse occurred more frequently in the humeral graft or proximal tibial graft than in the scapula. Davidson et al. and Jones et al. also reported high rates of bone resorption in proximal humeral ECIA [8, 11]. Bus et al. reported a high risk of fracture for proximal humeral and tibial allograft [14]. We think that the high incidence of bone resorption in the proximal humeral graft and the proximal tibial graft was associated with poor revascularization and/or difficult rigid fixation. The bone graft of the humeral head and tibial plateaus needs to be revascularized from the slim distal end of the bone, while the glenoid of the scapula can be revascularized from the wide end of the bone. In addition, rigid fixation of the proximal humeral graft with an adequate support of the spherical subchondral bone of the humeral head is considered to be impossible by any implant available today. We did not show an association between the fixation method and clinical outcome. However, two cases of distal femoral osteoarticular graft with rigid fixation with screws just below the articular surface supporting the subchondral bone did not demonstrate subchondral bone collapse in our series (Cases #22 and #23, Figure 5). Therefore, we think that rigid fixation of the entire length of the graft, including subchondral bone to the host bone, is a necessary requirement for the survival of the osteoarticular graft. Additionally, revascularization of the ECIA is needed to prevent bone resorption or collapse of the osteoarticular graft. Thus, if the vascularized fibula combined with the ECIA can support subchondral bone of the ECIA and revascularize the ECIA, the survival of the osteoarticular graft may be improved even in proximal humerus and proximal tibia.

In this study, functional outcome was not different among the graft sites, but it was related to the ISOLS radiographic scores as previously reported [11]. Thus, we need to prevent subchondral bone collapse or bone resorption by rigid graft fixation in order to achieve the maximum functional outcome. The functional outcomes in this study are comparable to those reported using other reconstruction methods. However, one limitation of this study is that the functional analysis was limited only to patients who still had the graft at the time of the survey.

**Figure 6:**
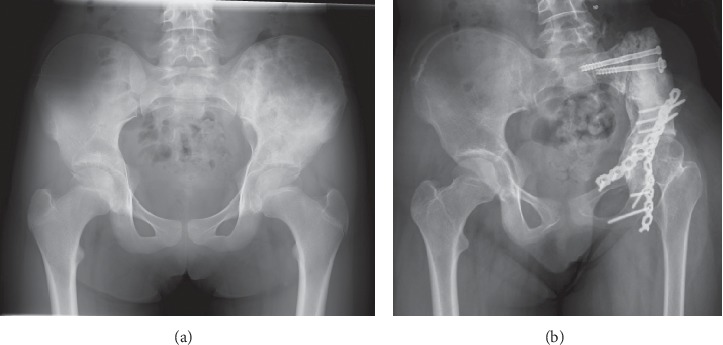
Case #19: 11-year-old male with Ewing sarcoma of the left pelvis. (a) Preoperative X-ray. (b) 6 years after radiograph of treatment with osteoarticular extracorporeal irradiated autograft. Osteonecrosis of the left femoral head occurred with acceptable functional results.

The advantage of the ECIA over endoprosthetic replacement or prosthetic composite graft is the following three points. First, it can adapt to any location or any shape of bone defect. Several reports about ECIA show acceptable outcomes in patients with bone malignancy in the scapula or pelvis [17,18]. In the present study, several cases of malignancy in the scapula or pelvis demonstrated good clinical outcomes (Figures 4 and 6). Acetabular and scapular flail surgery also often offers good function. However, ECIA is superior to flail surgery in that it can minimize leg length discrepancy and cosmetic deformity. Second, it does not damage the growth plate in the surrounding healthy bone. Limb salvage surgery in skeletally immature patients with bone sarcoma is a challenging issue. Surgical options for these patients include amputation, rotationplasty, expandable prosthesis, distraction osteogenesis, and recycled autografts or allografts. Recycled autografts or allografts can minimize leg discrepancy by preserving the growth plate of the surrounding bone. In our series, 4 of 6 skeletally immature patients with bone sarcoma around the knee still had ECIA at the time of the survey with acceptable leg discrepancy and functional outcomes (Figure 5). Finally, there are few late complications. In this study, removal of ECIA was required in 5 cases within 25 months, but there were no cases in which ECIA was removed after the 25th month (Figure 3). This is in contrast to the fact that megaprosthesis often needs to be removed long after surgery due to infection, loosening, and so on. Therefore, this procedure is considered to be one of the reasonable surgical options for patients with bone sarcoma in unusual location including the scapula or pelvis, or for skeletally immature patients who need long-term stability.

From our experiences written in this paper, we now prefer ECIA-prosthesis composite to osteoarticular ECIA for the bone tumor around the knee or proximal humerus in the adults. From 1988 to 2000, osteoarticular ECIA accounted for 50% and ECIA-prosthesis composite accounted for 10% among all ECIAs. However, from 2001 to 2014 osteoarticular ECIA decreased to 30% and ECIA prosthesis composite increased to 35%. The ratio of intercalary ECIA and hemicortical ECIA among all ECIAs did not change between the earlier period and the later period.

## 5. Conclusions

We do not recommend osteoarticular ECIA as a routine procedure due to the high risk of complication, which is comparable to that of osteoarticular frozen autograft. ECIA is considered to be one of the reasonable surgical options for patients with tumors for which reliable prostheses are not available such as the tumor arising in the scapula or pelvis or the tumor in skeletally immature patients.

## Figures and Tables

**Table 1:** Patient characteristics.

No.	Site	Age (years)	Sex	Tumor	Follow-up (months)	Patient status	Reason for reoperation	Salvage procedure	Graft removal (months)	Radiographic evaluation (total (%))	MSTS (total (%))	Leg length discrepancy
1	Scapula glenoid	47	M	Bone met (clear cell sarcoma)	5	DOD	—	—	—	32 (100)	NA^*∗*^	—
2	Scapula glenoid	61	M	Osteosarcoma	81	CDF	—	—	—	31 (96.9)	NA^*∗*^	—
3	Scapula glenoid	40	M	Chondrosarcoma	52	CDF	Wire protrusion	Wire removal	—	31 (96.9)	25(83.3)	—
4	Proximal humerus	22	F	Osteosarcoma	55	CDF	—	—	—	27 (84.4)	23 (76.7)	—
5	Proximal humerus	6	F	Osteosarcoma	106	NED	Fracture	ORIF	—	9 (28.1)	19 (63.3)	—
6	Proximal humerus	12	F	Osteosarcoma	191	CDF	IM rod protrusion	IM rod removal	—	16 (50.0)	26 (86.7)	—
7	Proximal humerus	58	M	Osteosarcoma	264	CDF	Infection	Debridement of soft tissue	—	25 (78.1)	23 (76.7)	—
8	Proximal humerus	14	F	Osteosarcoma	256	CDF	—	—	—	18 (56.3)	NA^*∗*^	—
9	Proximal humerus	49	F	Bone met (thyroid)	252	AWD	Screw protrusion	Screw removal	—	16 (50.0)	22 (73.3)	—
10	Proximal humerus	17	F	Ewing sarcoma	172	CDF	—	—	—	17 (53.1)	18 (60.0)	—
11	Proximal humerus	18	M	Osteosarcoma	125	CDF	—	—	—	17 (53.1)	23 (76.7)	—
12	Distal humerus	8	M	Mesenchymal chondrosarcoma	192	CDF	Infection	Removal of ECIA, fibula graft	13	NA	NA^*∗*^	—
13	Distal humerus	13	F	Osteosarcoma	152	CDF	—	—	—	19 (59.4)	23 (76.7)	—
14	Proximal ulna	67	M	Synovial sarcoma	82	DOD	—	—	—	32 (100)	NA^*∗*^	—
15	Proximal radius	25	M	Liposarcoma	67	CDF	—	—	—	22 (68.8)	23 (76.7)	—
16	Distal radius	14	F	Osteosarcoma	252	CDF	—	—	—	17 (53.1)	18 (60.0)	—
17	Metacarpal	43	M	Clear cell sarcoma	47	DOD	—	—	—	18 (56.3)	NA^*∗*^	—
18	Acetabulum	15	M	Myxoid liposarcoma	41	DOD	—	—	—	29 (90.6)	NA^*∗*^	—
19	Acetabulum	11	F	Ewing sarcoma	75	NED	Infection	Debridement of soft tissue, muscle cutaneous flap	—	29 (90.6)	22 (73.3)	—
20	Acetabulum	64	M	Chondrosarcoma	220	DOD	Infection	Debridement of soft tissue	—	8 (25.0)	21 (70.0)	—
21	Proximal femur	21	F	Osteosarcoma	57	DOD	Infection	Debridement of soft tissue	—	19 (59.4)	NA^*∗*^	—
22	Distal femur	6	M	Osteosarcoma	129	CDF	—	—	—	26 (81.3)	24 (80.0)	36 mm^*∗*^^*∗*^
23	Distal femur	13	M	Osteosarcoma	256	CDF	—	—	—	27 (84.4)	28 (93.3)	57 mm
24	Distal femur	13	M	Osteosarcoma	264	NED	Infection	Removal of ECIA, endoprosthetic replacement	14	NA	NA^*∗*^	NA^*∗*^
25	Proximal tibia	9	M	Osteosarcoma	47	DOD	Infection	Removal of ECIA	9	NA	NA^*∗*^	NA^*∗*^
26	Proximal tibia	20	M	Osteosarcoma	258	CDF	Infection	Removal of ECIA, prosthetic composite TKA	25	18 (56.3)	NA^*∗*^	—
27	Proximal tibia	17	M	Osteosarcoma	207	CDF	Degenerative change	Removal of ECIA, prosthetic composite TKA	16	16 (50.0)	NA^*∗*^	—
28	Proximal tibia	17	M	Liposarcoma	214	CDF	Infection	Debridement of soft tissue	—	15 (46.9)	16 (53.3)	—
29	Proximal tibia	8	F	Ewing sarcoma	103	CDF	Screw protrusion	Screw removal	—	20 (62.5)	23 (76.7)	32 mm
30	Proximal tibia	10	F	Osteosarcoma	97	CDF	—	—	—	18 (56.3)	23 (76.7)	40 mm
31	Distal tibia	26	F	Low-grade central osteosarcoma	134	CDF	—	—	—	28 (87.5)	23 (76.7)	—
32	Distal tibia	40	M	Osteosarcoma	90	CDF	—	—	—	15 (46.9)	18 (60.0)	—
33	Distal tibia	13	F	Osteosarcoma	46	CDF	—	—	—	22 (68.8)	24 (80.0)	—

^*∗*^Functional score or leg discrepancy was not recorded due to death, graft removal, or lost to follow-up. ^*∗*^^*∗*^Percutaneous epiphysiodesis using transphyseal screws of the contralateral distal femur and proximal tibia was performed.

**Table 2:** ISOLS radiographic evaluation form for osteoarticular graft.

	Fusion	Resorption	Fracture	Graft shortening	Fixation	Subluxation	Joint narrowing	Subchondral bone
Excellent = 4	Osteotomy line no longer visible	No resorption or geometric change Periosteal new bone formation	No fracture	No shortening	No change	No change	No change	No change
Good = 3	Fusion ≥75% of cortical thickness, osteotomy line still visible	Resorption <25% of cortical thickness and no fracture	Incomplete fracture	Shortening <2 cm	Minor change <10° lysis around screw/plate without failure	<0.5 cm	<2 mm	X-ray change but no collapse
Fair = 2	Fusion 25–75% of cortical thickness	Resorption 25–50% of cortical thickness and no fracture	Simple fracture without displacement	Shortening 2–4 cm	Bending >10° broken device not affecting graft soft tissue detachment	0.5–1 cm	2–4 mm	Partial collapse <1 cm
Poor = 1	No evidence of callus or fusion <25% of cortical thickness	Resorption >50% or type 1 fracture with resorption	Simple fracture with displacement or comminuted fracture	Shortening >4 cm	Failure of device with damage of graft	>1 cm	>4 mm	Partial collapse >1 cm or total collapse type 3 fracture

**Table 3:** Difference among the graft sites.

Site	Reoperation	Graft removal	Radiographic evaluation									MSTS (%)
			Fusion	Resorption	Fracture	Graft shortening	Fixation	Subluxation	Joint narrowing	Subchondral bone	Total (%)	
Scapula glenoid	1/3 (33%)	0/3	4.0	4.0	4.0	4.0	3.3	4.0	4.0	4.0	97.9	83.3
Proximal humerus	4/8 (50%)	0/8	2.8	2.1	3.6	2.6	2.6	1.4	1.4	1.6	56.6	73.3
Acetabulum	2/3 (67%)	0/3	2.7	2.3	3.0	3.0	3.0	3.0	2.3	2.7	68.6	71.7
Distal femur	1/3 (33%)	1/3 (33%)	3.5	3.0	4.0	4.0	3.5	3.0	2.5	3.0	82.8	80.0
Proximal tibia	5/6 (83%)	3/6 (50%)	3.6	2.0	3.4	2.4	3.0	1.0	1.0	1.0	54.5	68.9
Distal tibia	0/3 (0%)	0/3	3.0	2.7	3.3	3.0	4.0	2.7	1.3	1.7	67.7	72.2
Other locations	2/7 (29%)	1/7 (14%)	2.8	2.5	3.5	2.8	3.2	2.2	2.0	2.2	66.2	71.1
Total	15/33 (45%)	5/33 (15%)	3.1	2.5	3.5	2.9	3.1	2.1	1.9	2.1	66.4	73.8

**Table 4:** Comparative data with other published series of ECIA.

Author	Year	Patients	Graft type	Follow-up (months)	Complication	Function
Chen et al. [7]	2005	15	IC	71	33%	87% (Enneking)
Davidson et al. [8]	2005	50	Com, IC, OA	38	34%	77% (MSTS), 81%(TESS)
Krieg et al. [5]	2007	16	IC	50	NA	85% (MSTS), 94%(TESS)
Puri et al. [6].	2012	32	IC	34	45%	87% (MSTS)
Kotb and Mostafa [9]	2013	42	Com, IC, OA	54	12%	77% (MSTS), 81%(TESS)
Arpornchayanon et al. [10]	2013	30	Com, IC, OA	47	33%	80% (MSTS), 81%(TESS)
Jones et al. [11]	2017	113	Com, IC, OA	80	37%	79% (MSTS), 83%(TESS)
Wu et al. [12]	2018	79	Com, IC, OA	82	44%	NA
This study		33	OA	125	45%	73.8% (MSTS)

IC: intercalary graft; Com: autograft prosthetic composite graft; OA: osteoarticular graft.

## Data Availability

The datasets used in this study are not publicly available due to patient integrity but are available from the corresponding author on reasonable request.

## Acronyms

ECIA: Extracorporeal irradiated autograft
ISOLS: International Society of Limb Salvage
MSTS: Musculoskeletal Tumor Society.
